# Transient Increased Calcium and Calcitriol Requirements After Discontinuation of Human Synthetic Parathyroid Hormone 1-34 (hPTH 1-34) Replacement Therapy in Hypoparathyroidism

**DOI:** 10.1002/jbmr.2555

**Published:** 2015-08-03

**Authors:** Rachel I Gafni, Lori C Guthrie, Marilyn H Kelly, Beth A Brillante, C Michele Christie, James C Reynolds, Nancy A Yovetich, Robert James, Michael T Collins

**Affiliations:** 1 Skeletal Clinical Studies Unit (SCSU), Craniofacial and Skeletal Diseases Branch (CSDB), National Institute of Dental and Craniofacial Research (NIDCR), National Institutes of Health (NIH), Bethesda, MD, USA; 2 Department of Pediatrics, Kaiser Permanente, Springfield, VA USA; 3 Clinical Center (CC)/Nuclear Medicine Department (NMD), National Institutes of Health (NIH), Bethesda, MD, USA; 4 Rho, Inc., Chapel Hill, NC, USA

**Keywords:** PARATHYROID-RELATED DISORDERS, PTH, BIOCHEMICAL MARKERS OF BONE TURNOVER, DXA, HORMONE REPLACEMENT

## Abstract

Synthetic human PTH 1-34 (hPTH 1-34) replacement therapy in hypoparathyroidism maintains eucalcemia and converts quiescent bone to high-turnover bone. However, the skeletal and metabolic effects of drug discontinuation have not been reported. Nine subjects with hypoparathyroidism received subcutaneous injections of hPTH 1-34 two to three times daily for 19.8 to 61.3 months and then transitioned back to calcium and calcitriol. Biochemistries and bone mineral density (BMD) by dual-energy X-ray absorptiometry (DXA) were assessed at baseline, while on treatment, and at follow-up 3 to 12 months after drug discontinuation. Two subjects developed hypocalcemia when hPTH 1-34 was abruptly discontinued. Thus, to avoid hypocalcemia, subjects were slowly weaned from hPTH 1-34 over several weeks. When hPTH 1-34 was stopped, subjects were requiring two to three times pretreatment doses of calcitriol and calcium to maintain blood calcium levels. Doses were gradually reduced over many weeks until calcium levels were stable on doses similar to baseline. Bone-specific alkaline phosphatase (BSAP), N-telopeptide (NTX), and osteocalcin (OC) increased significantly with hPTH 1-34; at follow-up, BSAP and NTX had returned to baseline while OC was still slightly elevated. During treatment, BMD was unchanged at the hip and lateral spine but declined at the anterior-posterior (AP) spine, radius, and total body. During weaning, BMD increased, with the hip and lateral spine exceeding pre-hPTH 1-34 values and the whole body returning to baseline. AP spine was increased non-significantly compared to baseline at follow-up. hPTH 1-34 must be gradually weaned in hypoparathyroid patients with high doses of oral medications given to avoid hypocalcemia. The transient increased requirements accompanied by increased BMD after long-term hPTH 1-34 therapy suggest a reversal of the expanded remodeling space favoring bone formation as the skeleton returns to a low-turnover state, reminiscent of the hungry bone syndrome. Further study and close monitoring is required to ensure safe transition to conventional therapy and to elucidate the physiological mechanism of this phenomenon.

## Introduction

Hypoparathyroidism, the decreased production or secretion of parathyroid hormone (PTH), may be due to genetic abnormalities, autoimmune disease, infiltrative conditions, or a complication of neck surgery. Patients with hypoparathyroidism experience hypocalcemia, hyperphosphatemia, and inappropriately normal or frankly elevated urinary calcium excretion^(1)^ In the adult skeleton, hypoparathyroidism results in a low-turnover state with low or low-normal bone turnover markers,^(2)^ decreased tetracycline uptake and increased bone volume on iliac crest bone biopsies,^(3,4)^ and variable changes in bone mineral density (BMD) on dual-energy X-ray absorptiometry (DXA) and quantitative computed tomography (QCT).^(5–7)^

Human synthetic PTH 1-34 (hPTH 1-34) hormone replacement therapy has been shown to be effective for the management of hypocalcemia in hypoparathyroidism,^(8,9)^ while at the same time converting bone to a high bone turnover state. Studies of intermittent hPTH 1-34 treatment have demonstrated increases in bone turnover markers, increases in trabecular bone and cortical porosity on iliac crest bone biopsies,^(10)^ and variable changes in BMD by DXA, with bone loss sometimes noted at the distal 1/3 radius,^(8–10)^ suggesting that hPTH 1-34 replacement therapy has differential effects on trabecular and cortical bone. However, the effects of hPTH 1-34 discontinuation on mineral metabolism, bone turnover, and BMD have not been previously reported.

## Patients and Methods

### Patients

Nine patients (3 men, 6 women), mean age 37 ± 13.3 years, with hypoparathyroidism for more than 1 year, were referred to the National Institutes of Health (NIH) for inclusion in a study evaluating the effects of 5 years of hPTH 1-34 replacement therapy on the skeleton (Table 1 ). Subjects were excluded from enrollment if they had significant liver or kidney disease (estimated glomerular filtration rate [eGFR] < 25 mL/min/1.73 m^2^), chronic disease known to affect mineral metabolism, a history of chronic steroid or bisphosphonates use, active thyroid cancer, or pregnancy. All female subjects were premenopausal at the start of hPTH 1-34 therapy. Mineral metabolism, bone density, and BMD results for the first 18 months of hPTH 1-34 therapy in 4 subjects have been previously published.^(10)^ One additional treated subject, subject #4 from that report, is not included in this analysis because she was pregnant during the time of hPTH 1-34 discontinuation. Additionally, data from only 8 subjects are included in this analysis because 1 subject did not undergo hPTH 1-34 weaning. The Institutional Review Board of the National Institute of Dental and Craniofacial Research (NIDCR) approved this protocol. Written informed consent was obtained from adult subjects or the parents of adolescent subjects; written assent was obtained from the adolescents.

### Therapy of hypoparathyroidism

Following an optimization period on conventional therapy, calcitriol was discontinued and subjects were started on twice daily (BID) (*n* = 6) or thrice daily (TID) (*n* = 3) subcutaneous injections of synthetic human hPTH 1-34, beginning the morning after the last dose of calcitriol. The study was initially designed to compare twice-daily with thrice-daily dosing, with subjects being randomly assigned to a treatment group. However, with a change in principal investigator, the decision was made to focus on the skeleton as the primary endpoint, rather than the dosing regimen. The design was therefore changed such that subjects on thrice-daily PTH were switched to twice-daily and all new subjects were started on twice-daily injections. Thus, only 1 subject was receiving thrice-daily injections at the time of hPTH 1-34 discontinuation. However, this subject was excluded from analysis because his hPTH 1-34 dose was not weaned (subject #9).

hPTH 1-34 lyophilized powder was purchased from Bachem, Inc. (Torrance, CA, USA) and then prepared and packaged by the NIH Pharmaceutical Development Service as described.^(11)^ The mean starting dose was 0.45 ± 0.09 μg/kg/day in the BID subjects and 0.37 ± 0.17 μg/kg/day in the TID subjects. However, the doses were rapidly adjusted as needed to maintain the serum calcium level at or slightly less than the lower limit of normal (7.6 to 9 mg/dL, 1.9 to 2.25 mmol/L). Calcium intake, through diet or supplements while on hPTH 1-34, was targeted at 1000 to 2000 mg/day; patients with low or irregular dietary calcium intake also took calcium supplements. Subjects found to be vitamin D-deficient were treated with ergocalciferol or cholecalciferol to maintain the 25-hydroxy vitamin D level above 62.5 nmol/L (25 ng/mL).

### Monitoring

Subjects were evaluated at the Clinical Center of the NIH (Bethesda, MD, USA) every 6 months. Testing included measurement of routine blood and urine biochemistries, 25-hydroxy vitamin D, bone-specific alkaline phosphatase (BSAP) (immunoenzymatic assay; Mayo Medical Labs, Rochester, MN, USA), osteocalcin (OC) (electrochemiluminescence; Clinical Center, NIH, Bethesda, MD, USA), and 24-hour urine collagen cross-linked N-telopeptide (NTX) (Vitros ECi competitive assay; Mayo Medical Labs). Bone density was assessed by DXA (Hologic, Bedford, MA, USA) every 6 months while on hPTH 1-34 and at a follow-up visit 6 to 12 months after the initiation of hPTH 1-34 weaning. Daily spine phantom BMD values evaluated for 6-month periods had a coefficient of variation of less than 0.4%. Percent coefficient of variation from precision testing of each site was as follows: lumbar spine (1.1%), total body (1.1%), 1/3 distal radius (1.6%), femoral neck (1.8%), and total hip (0.9%). To normalize for the variation in age and gender of the subjects, *Z*-scores were calculated using published normative databases for the adolescent^(12)^ and the Hologic-provided normative database for adults. As normative data for the lateral spine are not available for men or children, *Z*-scores were not calculated for this site. Between visits to the NIH, blood and urine mineral metabolism was monitored per protocol at the subjects’ local laboratories and therapy was adjusted to maintain the serum calcium level at or slightly below the lower limit of normal (7.6 to 9 mg/dL, 1.9 to 2.25 mmol/L).

### Weaning of hPTH 1-34

In the first 2 subjects to be discontinued, calcitriol and calcium were restarted at their pre-hPTH 1-34 (baseline) doses for 2 to 3 days without changing the dose of hPTH 1-34. hPTH 1-34 was then decreased abruptly over 2 to 3 days, resulting in significant symptomatic hypocalcemia requiring high doses of calcium and calcitriol with reinitiation and gradual tapering of hPTH 1-34 in subject #1; the other subject (#9) was not restarted on hPTH 1-34 and is excluded from this analysis. Based on this experience, for subsequent subjects, calcitriol and calcium supplements were restarted at doses two to three times higher than baseline. Calcium levels were then monitored one to two times per week with gradual decreases in hPTH 1-34 and adjustments in calcium and calcitriol made to maintain serum calcium within the aforementioned target range. Following the final dose of hPTH 1-34, subjects were assessed every 1 to 2 weeks with adjustment of calcium and calcitriol doses until calcium levels stabilized. Once stable, calcium levels were measured every 1 to 3 months until their return to NIH for the post hPTH 1-34 follow-up visit.

### Statistical analysis

Unless otherwise stated, results are presented as mean ± SD. Mixed models repeated measures analysis (PROC MIXED, SAS version 9.3; SAS Institute, Cary, NC, USA) were performed on outcome measures at three time points: prior to hPTH 1-34 (baseline), start of hPTH 1-34 weaning, and follow-up. To normalize data and stabilize the within-treatment variance, bone turnover markers, calcium, and phosphorus levels were log-transformed and geometric means with 95% confidence limits are presented. For BMD measurements, both nontransformed absolute BMD and BMD *Z*-scores were analyzed, with the exception of the lateral spine *Z*-scores because there is no normative data available for adolescents or men. Pairwise post hoc contrasts were made to compare the effects of treatment based on the study time-points. Significance was assumed at a *p* < 0.05. When the repeated measures overall effects of study time-point were not significant, Bonferroni corrections were used to adjust the pairwise contrasts for the three multiple comparisons within an outcome measure, with significance assumed for a *p* < 0.0167.

## Results

Prior to initiation of hPTH 1-34, subjects were managed conventionally with oral calcitriol (0.69 ± 0.22 μg/day) and elemental calcium (1425 ± 656 mg/day). The mean duration of hPTH 1-34 therapy prior to weaning was 46 ± 18 months. Five subjects completed the full 5 years of therapy per the protocol. Four subjects discontinued hPTH 1-34 early; 1 because of bone pain (subject#1) and 3 because of a decline in radial BMD *T*-score or *Z*-score to < −2 (subjects #7, #8, and #9). In the 2 months prior to initiation of weaning, the mean dose of calcium supplementation was 514 ± 565 mg/day; no subjects were taking calcitriol. The mean dose of hPTH 1-34 therapy at the initiation of weaning was 0.54 ± 0.24 μg/kg/day.

### Weaning phase

Subjects #1 and #9 were the first subjects to discontinue hPTH 1-34 therapy. They were restarted on calcitriol and calcium at one to two times their pre-hPTH 1-34 doses and hPTH 1-34 was rapidly decreased and discontinued over 2 to 3 days. Three days after stopping hPTH 1-34, subject #9 experienced hypocalcemia with a calcium level of 6.3 mg/dL that was rapidly corrected with an increase in calcium and calcitriol; hPTH 1-34 was not restarted in this subject. Subject #1 also experienced symptomatic hypocalcemia (serum calcium 6.6 mg/dL); in this case, hPTH 1-34 was restarted and the subject was weaned slowly. As a result, the protocol was revised such that all patients would be weaned gradually off hPTH 1-34 as tolerated, based on frequent monitoring and symptomatology.

**Fig. 1.**
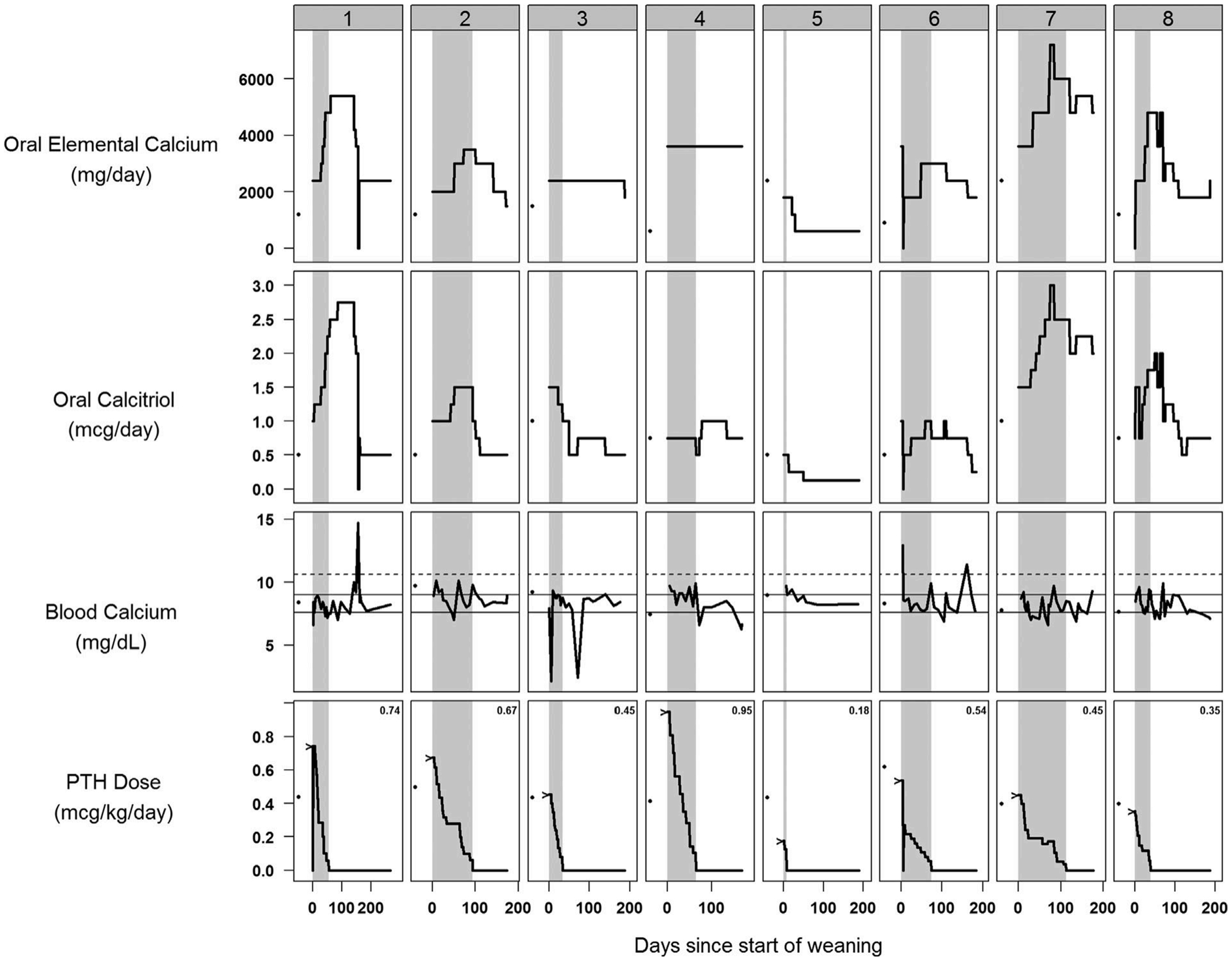
Elemental calcium dose, calcitriol dose, blood calcium, and hPTH 1-34 dose during and after PTH weaning for subjects #1 to #8. Shaded area indicates the weaning period. Horizontal lines on serum calcium plots indicate upper and lower limits of target range (solid lines, 7.6 to 9 mg/dL) and upper limit of normal range (dashed line, 10.5 mg/dL). Lower limit of normal range in euparathyroid individuals is 8.6 mg/dL (not shown). Baseline values at the start of hPTH 1-34 therapy are indicated using a dot. hPTH 1-34 doses at the start of weaning are indicated with a caret and text. To convert calcium levels to mmol/L, divide by 4.

Excluding subject #9, the mean duration of hPTH 1-34 therapy during the weaning period was 60 ± 34 days. Elemental calcium, calcitriol, and hPTH 1-34 doses required to maintain serum calcium levels within the target range are presented in Fig. 1. When hPTH 1-34 was discontinued, subjects were requiring two to three times their pre-hPTH 1-34 doses of calcitriol (1.28 ± 0.78 μg/day) and elemental calcium (3737 ± 1391 mg/day). High doses were needed for several more weeks in some subjects and were gradually reduced until calcium levels were stable. Of note, subject #1 developed acute hypercalcemia (14.7 mg/dL, 3.67 mmol/L) 3 months after her last dose of hPTH despite weekly blood monitoring and adjustment of calcium and calcitriol, suggesting an abrupt return to her baseline low-turnover state. By the follow-up visit, the mean doses for the group were not significantly different from pre-hPTH 1-34 levels: calcitriol 0.67 ± 0.22 μg/day and elemental calcium 2363 ± 1307 mg/day. Serum calcium and phosphorus measurements were not significantly different at baseline, start of weaning, or follow-up (Table 2).

### Bone markers

**Fig. 2.**
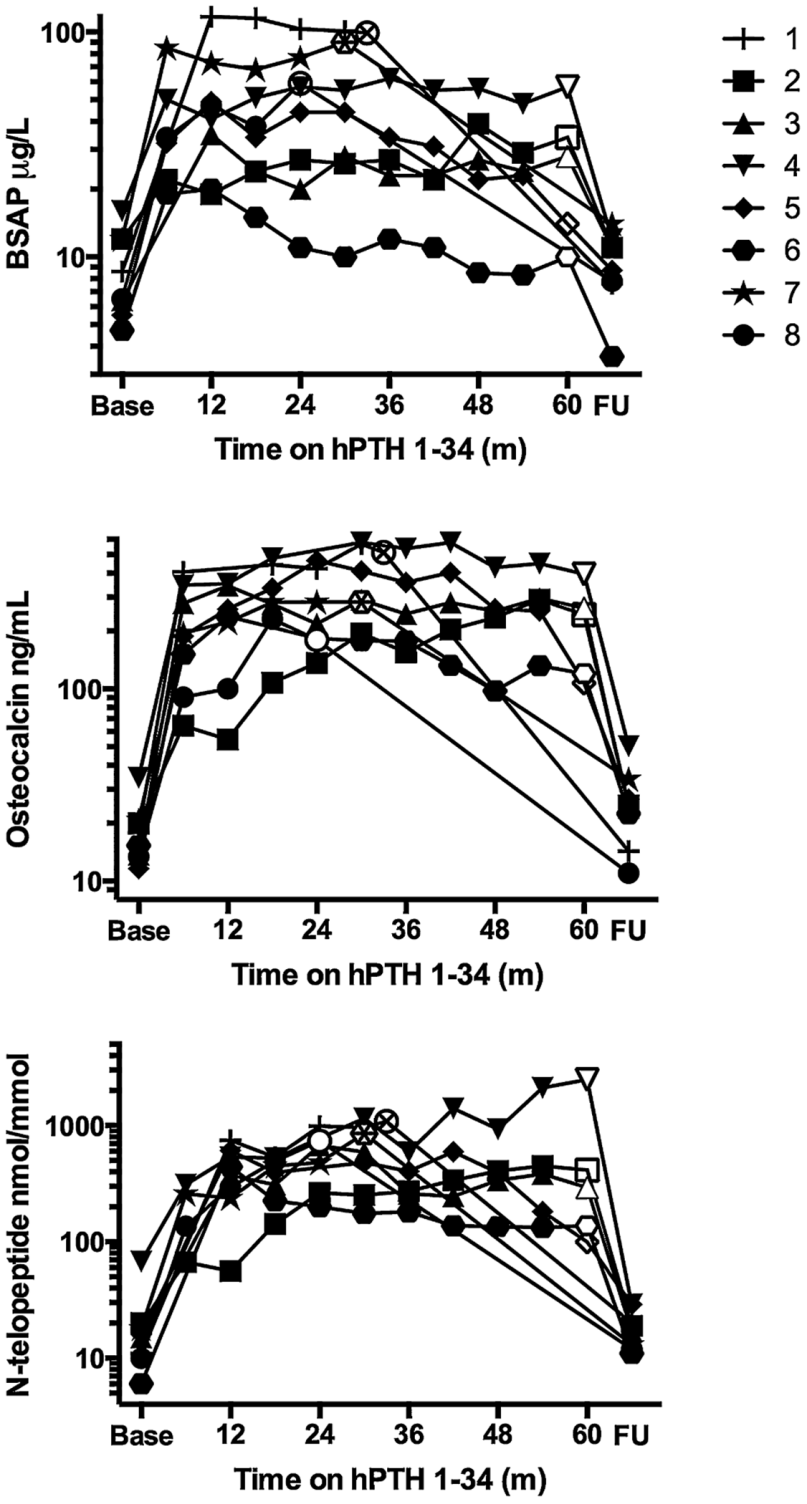
Bone-specific alkaline phosphatase, osteocalcin, and collagen cross-linked N-telopeptide levels for subjects #1 to #8 prior to hPTH 1-34 therapy (Base), during therapy, and at FU visit. White symbols indicate subjects’ values at the start of hPTH 1-34 weaning. Normal values are as follows: BSAP, 0 to 14 pg/L (premenopausal women), 0 to 20 pg/L (men), 7 to 41 pg/L (14- to 18-year-old female); OC, 9 to 40ng/mL (adults); NTX, 55 to 378 BCE/mmol Cr (14- to 18-year-old girls), 19 to 63 BCE/mmol Cr (women), 21 to 66 BCE/mmol Cr (men). FU = follow-up; BSAP = bonespecific alkaline phosphatase; OC = osteocalcin; NTX = collagen crosslinked N-telopeptide; BCE = bone collagen equivalent.

At baseline, bone turnover markers were normal or low, as expected for hypoparathyroidism (Table 2). hPTH-1-34 therapy significantly increased bone resorption and bone formation markers, which remained persistently elevated for the duration of therapy (Fig. 2). At follow-up, BSAP and NTX had returned to baseline levels, whereas OC was still slightly elevated and statistically different from baseline.

### Bone density

**Fig. 3.**
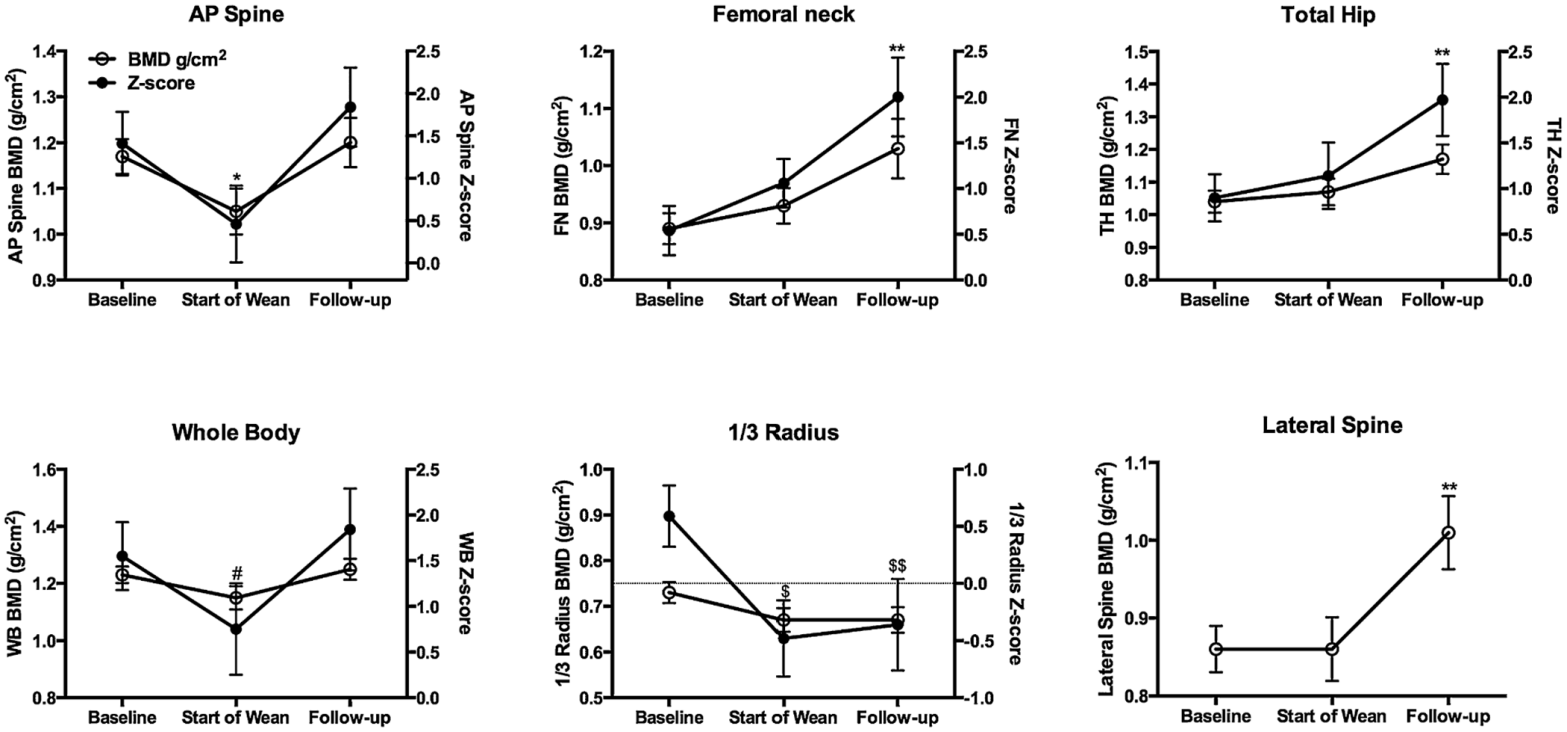
Areal BMD (g/cm^2^) and BMD Z-scores (SDs) prior to start of hPTH 1-34 (baseline), at start of weaning, and at FU visit. Data are presented as mean ±SE. (Left) y axes and open circles correspond to areal BMD. (Right) *y* axes and closed circles represent *Z*-scores. *Z*-scores are not included for the lateral lumbar spine as normative data for men are not available. Footnotes for significance apply to both areal BMD and BMD *Z*-score except where indicated. *p < 0.05 compared to baseline and FU; **p < 0.05 compared to baseline and start of wean; #*p* < 0.05 compared to baseline and FU (BMD only); $*p* < 0.01 compared to baseline; $$*p* < 0.05 compared to baseline (BMD only). BMD = bone mineral density; FU = follow-up; AP = anterior-posterior; FN = femoral neck; TH = total hip; WB = whole body.

Effects of hPTH 1-34 therapy on BMD by DXA varied at the different sites (Fig. 3). At the start of weaning, BMD and BMD *Z*-scores were significantly lower than baseline for the anterior-posterior (AP) spine and radius, but unchanged for the lateral spine, femoral neck, and total hip. For the whole body, absolute BMD was significantly decreased but the decrease in BMD *Z*-score was not significant. There were no significant changes with hPTH 1-34 treatment at the lateral spine, femoral neck, or total hip. At the follow-up visit, BMD and BMD *Z*-scores had increased at all sites compared to the start of weaning except for the distal radius, where only the *Z*-score increased. These increases were significant at the radius, AP spine, and whole body, where levels returned to baseline values, and the femoral neck, total hip, and lateral spine, where increases were dramatic and exceeded baseline values.

## Discussion

In patients with hypoparathyroidism treated with full replacement intermittent subcutaneous hPTH 1-34 for 18 to 60 months, without calcitriol, hPTH 1-34 could not be discontinued abruptly. Rather, high doses of calcium and calcitriol were required while hPTH 1-34 was slowly tapered to avoid hypocalcemia and maintain serum calcium levels within the target range. Furthermore, even after hPTH 1-34 was discontinued, significantly higher than maintenance calcium and calcitriol doses were necessary for several additional weeks until the subjects ultimately returned to their baseline, pre-hPTH 1-34 low-turnover state. Bone turnover markers were markedly elevated during hPTH 1-34 therapy. BSAP and NTX returned to low/low-normal values by 6 to 12 months after the initiation of the hPTH 1-34 wean; however, osteocalcin was still slightly higher than pre-hPTH 1-34 values. DXA scans performed at the follow-up visit revealed marked increases in BMD, suggesting that a slower decline in bone formation compared to bone resorption, along with concurrent high doses of calcium and calcitriol administered during the wean, resulted in increased skeletal calcium deposition.

PTH has paradoxical effects on the skeleton. Persistently high PTH, as seen in primary hyperparathyroidism, stimulates osteoclastic bone resorption, leading to increased remodeling space, hypercalcemia, and osteoporosis.^(13)^ When assessed by backscatter electron microscopy, the mean age and mineralization density of hyperparathyroid bone is decreased compared to controls, consistent with an increased bone turnover state; these parameters revert to normal with correction of hyperparathyroidism.^(14)^ When PTH is used intermittently as a treatment for osteoporosis, it similarly stimulates bone turnover increasing the amount of younger, less mineralized bone.^(15)^ In contrast to hyperparathyroidism, however, this increased turnover seen with intermittent administration appears to favor anabolism, rather than catabolism, stimulating osteoblast progenitors,^(16,17)^ inhibiting osteoblast apoptosis^(18)^ and increasing bone density at the lumbar spine and hip while decreasing BMD at the distal radius.^(19,20)^ When PTH therapy for osteoporosis is discontinued, spinal and hip gains in BMD are gradually lost and radial BMD increases.^(20)^ Patients with hypoparathyroidism have decreased bone formation rates and remodeling activation frequency in the absence of PTH;^(3)^ overtime they develop increased cancellous and cortical bone mass,^(4,21)^ despite hypocalcemia. Although less well studied, the mineralization density of hypoparathyroid bone was similar to controls in an adult woman with idiopathic hypoparathyroidism,^(22)^ suggesting that the increased bone mass in hypoparathyroidism is due to an overall increase in bone volume in hypoparathyroidism, and not an increase in mineralization.

Studies of hPTH 1-34 and recombinant human PTH 1-84 (rhPTH 1-84) treatment in hypoparathyroidism have demonstrated significant increases in bone remodeling, with markers of bone turnover exceeding the upper limit of normal and remaining persistently elevated for the duration of treatment.^(8,10,23,24)^ Effects on the skeleton assessed by iliac crest biopsies, DXA, and quantitative computed tomography have been variable, depending upon the drug dose and regimen used. In a small pilot study treating 5 subjects with full replacement therapy for 1 year, we saw dramatic increases in trabecular bone volume and intratrabecular tunneling with increased cortical porosity; BMD *Z*-scores did not change at the lumbar spine but decreased at the distal radius.^(10)^ These findings suggested that hPTH 1-34 replacement therapy had a differential effect on cortical versus trabecular bone. Uncontrolled studies with rhPTH 1-84 therapy have similarly shown increased trabecular bone, intratrabecular tunneling, and cortical porosity.^(25,26)^ In a 6-month placebo-controlled rhPTH 1-84 study, where subjects received relatively high doses and were frequently hypercalcemie, trabecular volume decreased with a trend toward increasing cortical porosity.^(27)^ In that study, there was a decrease in spinal areal BMD (aBMD) yet an increase in spinal volumetric BMD (vBMD), also supporting the observation that PTH therapy affects cortical and trabecular bone differentially. In 2013, Mannstadt and colleagues^(28)^ showed in a 6-month placebo-controlled study that the hypocalcemia could be effectively managed with variable doses of rhPTH 1-84, with additional calcitriol as needed; however, skeletal endpoints were not included in this report. This study did note that, during the first 4 weeks after study drug discontinuation, hypocalcemic events were reported in 31% of rhPTH 1-84-treated patients (32 events) compared with only 9% of the placebo-treated patients (four events).

The high requirements for calcium and calcitriol during the hPTH 1-34 taper in this study are reminiscent of the “hungry bone syndrome,” a condition of prolonged hypocalcaemia sometimes observed after parathyroid surgery in patients with hyperparathyroidism. Although the mechanism is not well-understood, hungry bone syndrome after parathyroidectomy is thought to be due to the abrupt cessation of PTH-mediated bone resorption despite continued increased bone formation; calcium is shunted into the skeleton resulting in postoperative hypocalcemia, increased calcium and vitamin D requirements, and consequent gains in bone mass.^(29)^ The risk of postoperative hungry bone syndrome appears to associated with older age, large parathyroid adenomas,^(30)^ and preexisting radiologic evidence of hyperparathyroid bone disease.^(31,32)^ Several studies, but not all, have reported an increased risk correlating with higher preoperative levels of PTH, calcium, and alkaline phosphatase.^(30–33)^ The duration of hungry bone syndrome is variable and has been reported to be as long as 9 months. Although the hypoparathyroid subjects in our study were not hypercalcémie while receiving hPTH 1-34 therapy, their bone turnover markers increased and lumbar spine and forearm BMD decreased while on treatment, consistent with a mixed hypoparathyroid/hyperparathyroid state. In the 2 patients in whom hPTH 1-34 was discontinued abruptly, severe hypocalcemia developed, likely due to the halting of bone resorption in the face of continued bone formation. Thus, gradual weaning of hPTH 1-34 with high doses of calcium and calcitriol were required, resulting in increased BMD. Of note, the return to the baseline low-turnover state can also be quite abrupt, as was seen in subject #1 who became acutely hypercalcemie despite frequent monitoring and medication adjustment.

This study is limited by its lack of a control group, small size, heterogeneous patient population, and variable duration of therapy. Despite these limitations, the magnitude of increased calcium and calcitriol requirements to prevent hypocalcemia during weaning, coupled with the dramatic increase in BMD seen at the follow-up visit suggest that this is a true effect of PTH therapy discontinuation in hypoparathyroidism.

This is the first study to detail the effects of PTH therapy discontinuation in a hypoparathyroid population. The high doses of calcium and calcitriol required during hPTH 1-34 weaning and the subsequent increased BMD suggest that, while overall bone turnover is declining and the remodeling space is shrinking, bone formation exceeds bone resorption for weeks to months after hPTH 1-34 discontinuation as the skeleton reverts from a high-turnover to a low-turnover state. To our knowledge, this phenomenon has not been studied in rhPTH 1-84, which has recently been U.S. Food and Drug Administration (FDA)-approved for the long-term treatment of hypoparathyroidism, although an increased incidence of hypocalcemic events after drug cessation was briefly noted by Mannstadt and colleagues.^(28)^ Given the similar effects of PTH 1-34 and 1-84 on bone turnover markers, bone densitometry, and iliac crest bone biopsies, it is reasonable to assume that this predominance of bone formation over resorption coupled with increased oral supplementation demands could also occur with discontinuation of rhPTH 1-84. Further study and close monitoring is necessary to ensure safe transition from PTH to conventional therapy, as well as to further elucidate the physiological mechanism of this phenomenon.

## Figures and Tables

**Table 1.** Subject Characteristics

Subject#	Age at start of hPTH 1-34 (years)	Gender	Diagnosis	Disease duration (years)	Duration of hPTH 1-34 therapy (months)^a^	hPTH 1-34 regimen
1	39	F	PS	2	33	TID→BID
2	36	F	22q11	36	60	TID→BID
3	46	F	PS	3	60	BID
4	15	F	22q11	15	60	BID
5	49	F	PS	3	60	BID
6	39	M	CaSR	39	60	BID
7	50	M	CaSR	50	30	BID
8	45	F	PS	4	24	BID
9^b^	15	M	?HDR	15	20	TID

hPTH 1-34 = human synthetic parathyroid hormone 1-34; F = female; PS = postsurgical; TID = three times per day; BID = two times per day; 22q11 del = deletion at chromosome 22q11 locus; M = male; CaSR = activating mutation of the calcium-sensing receptor; HDR, probable hypoparathyroidism-deafness-renal syndrome, genotyping not performed.

a Prior to start of wean.

b Subject #9 was never transitioned to BID dosing and was not weaned from hPTH 1-34 and was therefore excluded from analysis.

**Table 2.** Bone and Mineral Markers

	Baseline		Start of wean		Follow-up	
	Mean	95% CL	Mean	95% CL	Mean	95% CL
Calcium (mg/dL)	8.38	7.74–9.07	8.47	8.1–8.86	8.02	7.29–8.81
Phosphorus (mg/dL)	5.18	4.76–5.63	4.51	4.09–4.97	4.52	4.01–5.10
BSAP (μg/L)	8.2	5.7–11.9	37.6^a^	18.7–75.7	8.9^b^	6.2–12.6
OC (ng/mL)	15.7	12.0–20.6	232.5^a^	147.5–366.5	23.6^b,c^	15.9–35
NTX (BCE/mmol Cr)	14.9	8.5–26.2	486.5^a^	177.2–1335.5	17.8^b^	12.2–26.1

Data presented as geometric mean with 95% CL. Statistical significance adjusted for multiple comparisons when appropriate. Significant comparisons have their *p* values indicated with footnotes. Normal values are as follows: BSAP, 0 to 14 μg/L (premenopausal women), 0 to 20 μg/L (men), 7 to 41 μg/L (14- to 18-year-old female); OC, 9 to 40ng/mL (adults); NTX, 55 to 378 BCE/mmol Cr (14- to 18-year-old girls), 19 to 63 BCE/mmol Cr (women), 21 to 66 BCE/mmol Cr (men). Target range for serum calcium: 7.6 to 9 mg/dL (1.9 to 2.25 mmol/L), normal range for serum phosphorus: 2.5 to 4.5 mg/dL (0.8 to 1.45 mmol/L). Conversion to mmol/L: calcium divide by 4; phosphorus divide by 3.1.

CL = confidence limits; BSAP = bone-specific alkaline phosphatase; OC = osteocalcin; NTX = collagen cross-linked N-telopeptide; BCE = bone collagen equivalents.

a *p* < 0.0001 compared to baseline.

b *p* < 0.0001 compared to start of wean.

c *p* <0.05 compared to baseline.
